# A case of left ventricular free wall rupture after insertion of an IMPELLA® left ventricular assist device diagnosed by transesophageal echocardiography

**DOI:** 10.1186/s40981-021-00444-w

**Published:** 2021-04-26

**Authors:** Akito Mizuno, Shuji Kawamoto, Shuji Uda, Kenichiro Tatsumi, Chikashi Takeda, Tomoharu Tanaka, Kazuhiko Fukuda

**Affiliations:** Department of Anesthesia, Kyoto University Hospital, 54, Shogoinkawahara-cho, Sakyo-ku, Kyoto, 606-8507 Japan

**Keywords:** IMPELLA®, Left ventricular mechanical support, Myocardial infarction, Left ventricular perforation, Transesophageal echocardiography, Cardiac tamponade

## Abstract

**Background:**

The IMPELLA® is a minimally invasive left ventricular assist device. We report a case in which transesophageal echocardiography (TEE) was useful in diagnosis of left ventricular rupture after IMPELLA® insertion.

**Case presentation:**

A 75-year-old man presented to the emergency room with chest pain and underwent percutaneous coronary intervention for 100% stenosis of the left anterior descending branch #7. An IMPELLA® was inserted to stabilize the circulation, but hypotension persisted. Transthoracic echocardiography revealed increased pericardial effusion and suspicion of free wall left ventricular rupture, leading to emergency surgery. TEE revealed the IMPELLA® straying into the left ventricle apical wall and cardiac tamponade. Hemorrhage was observed from the thinning free wall and the tip of the IMPELLA® was palpable. The IMPELLA® was removed and the left ventricular wall was repaired.

**Conclusions:**

The IMPELLA® requires implantation of the tip in the left ventricle, but it should be noted that a fragile ventricular wall can be easily perforated.

## Background

The IMPELLA® is a minimally invasive left ventricular assist device that can be placed by catheterization. It has been shown to be effective and safe in patients with cardiogenic shock [1]. Left ventricular free wall rupture with IMPELLA® insertion has been reported in only three previous cases worldwide [2–4]. Here, we report a case of left ventricular free wall rupture after insertion of an IMPELLA® that was diagnosed by transesophageal echocardiography (TEE).

## Case presentation

A 75-year-old male (height, 175.6 cm; weight, 91.3 kg; body surface area, 2.028 m^2^) was transported to our hospital with a chief complaint of chest and back pain. An electrocardiogram showed ST-segment elevation in V1-4 inductions. Transthoracic echocardiography (TTE) showed severe wall motion abnormalities in the anterior wall, an ejection fraction of about 40%, and pericardial fluid volume within physiological limits with a maximum thickness of 4 mm. An intra-aortic balloon pump (IABP) was inserted for circulatory support, but the hypotension was still prolonged, leading to the introduction of a percutaneous cardiopulmonary support system (PCPS). The flow rate of 3.8 L/min, 3700 rpm and the arterial blood pressure of 120/70 mmHg was obtained at the start using 20 Fr for the arterial cannula and 24 Fr for the venous cannula. Percutaneous coronary intervention (PCI) was performed under PCPS and IABP support for 100% stenosis of the left anterior descending branch #7. However, the flow of PCPS decreased to about 1 L/min and the arterial blood pressure dropped to 70/50 mmHg, probably because of volume loss and vasopressor use, resulting in a blood transfusion. The IABP was replaced with an IMPELLA®, a ventricular support catheter, because the IABP was not thought to contribute to the improvement of circulatory dynamics in the insufficient systemic blood flow, and we expected to reduce the left ventricular load caused by the retrograde blood delivery of PCPS.

**Fig. 1:**
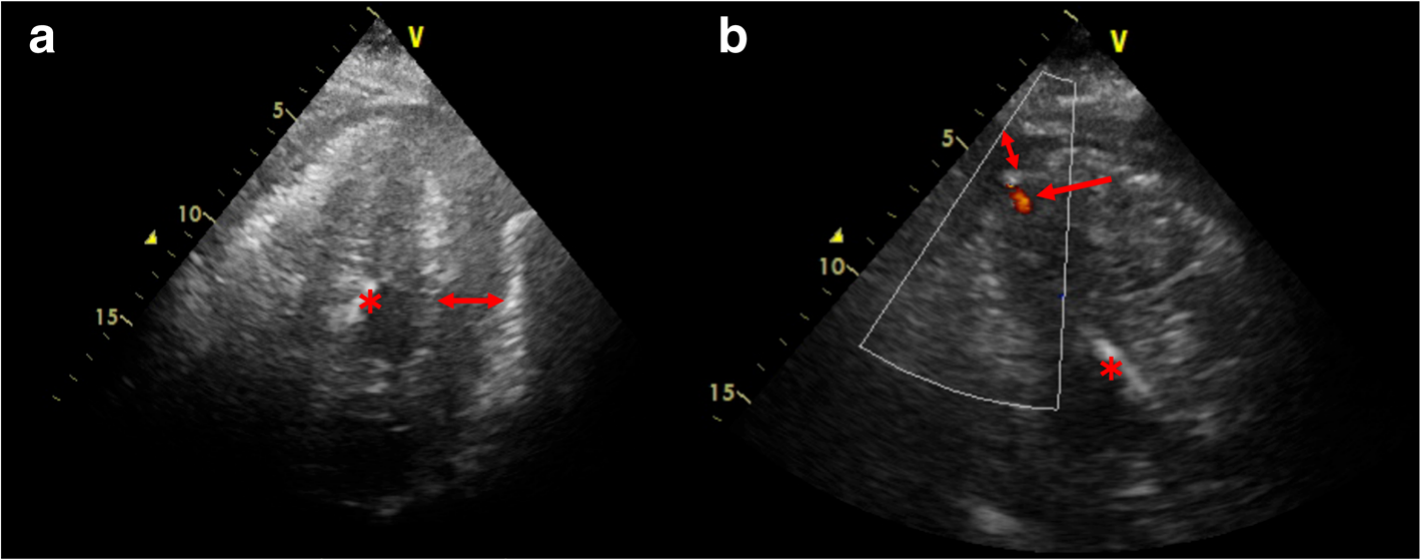
Apical four chamber view (**a**) and apical long-axis view (**b**) by transthoracic echocardiography. Note the pericardial effusion (double arrow) and the abnormal flow on apex of the left ventricle (small arrow), part of the IMPELLA® (asterisk). The tip of the IMPELLA® is not depicted

**Fig. 2:**
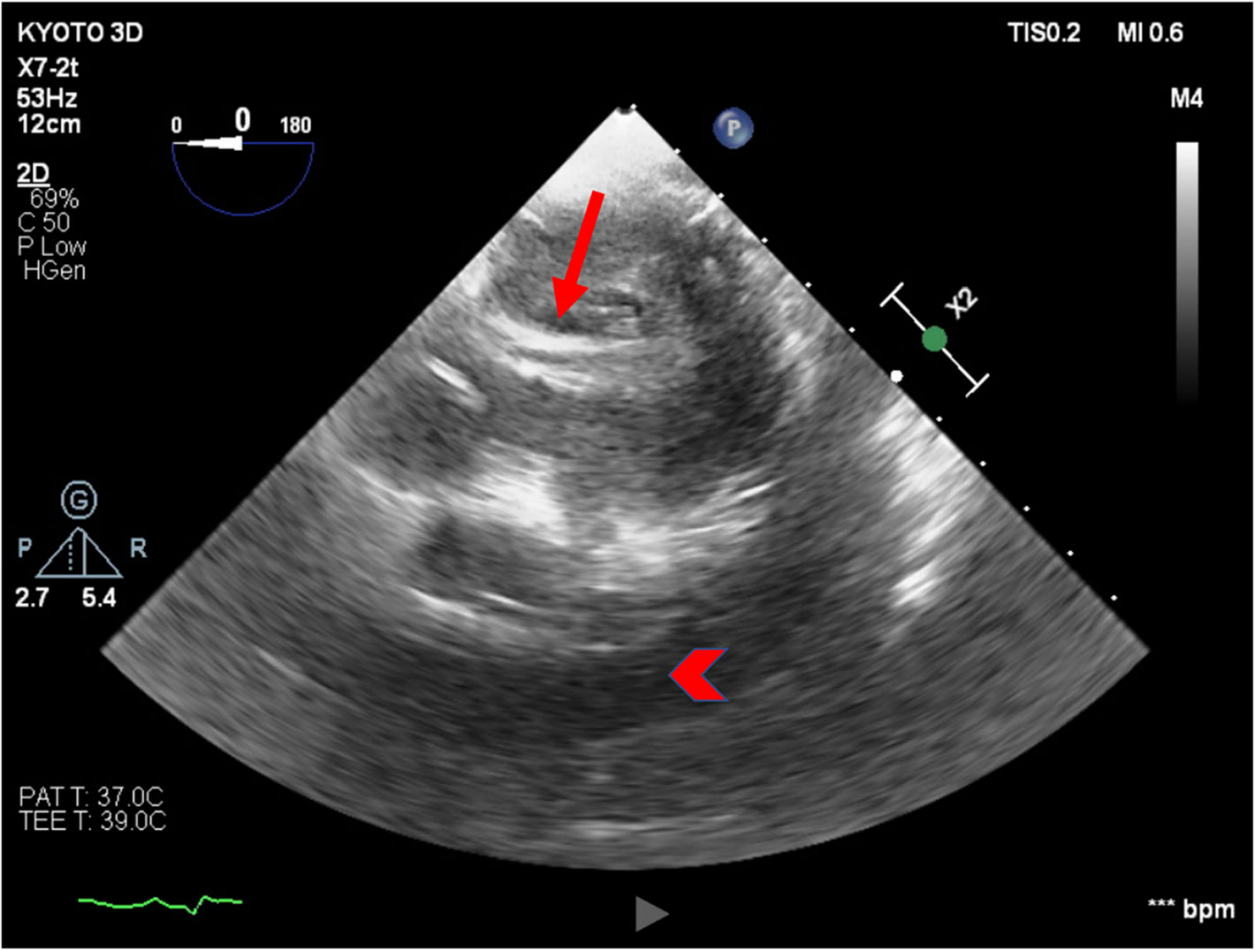
Transgastric apical short-axis view by transesophageal echocardiography. Note the massive pericardial effusion (arrow head) and, the part of the IMPELLA® (small arrow)

**Fig. 3:**
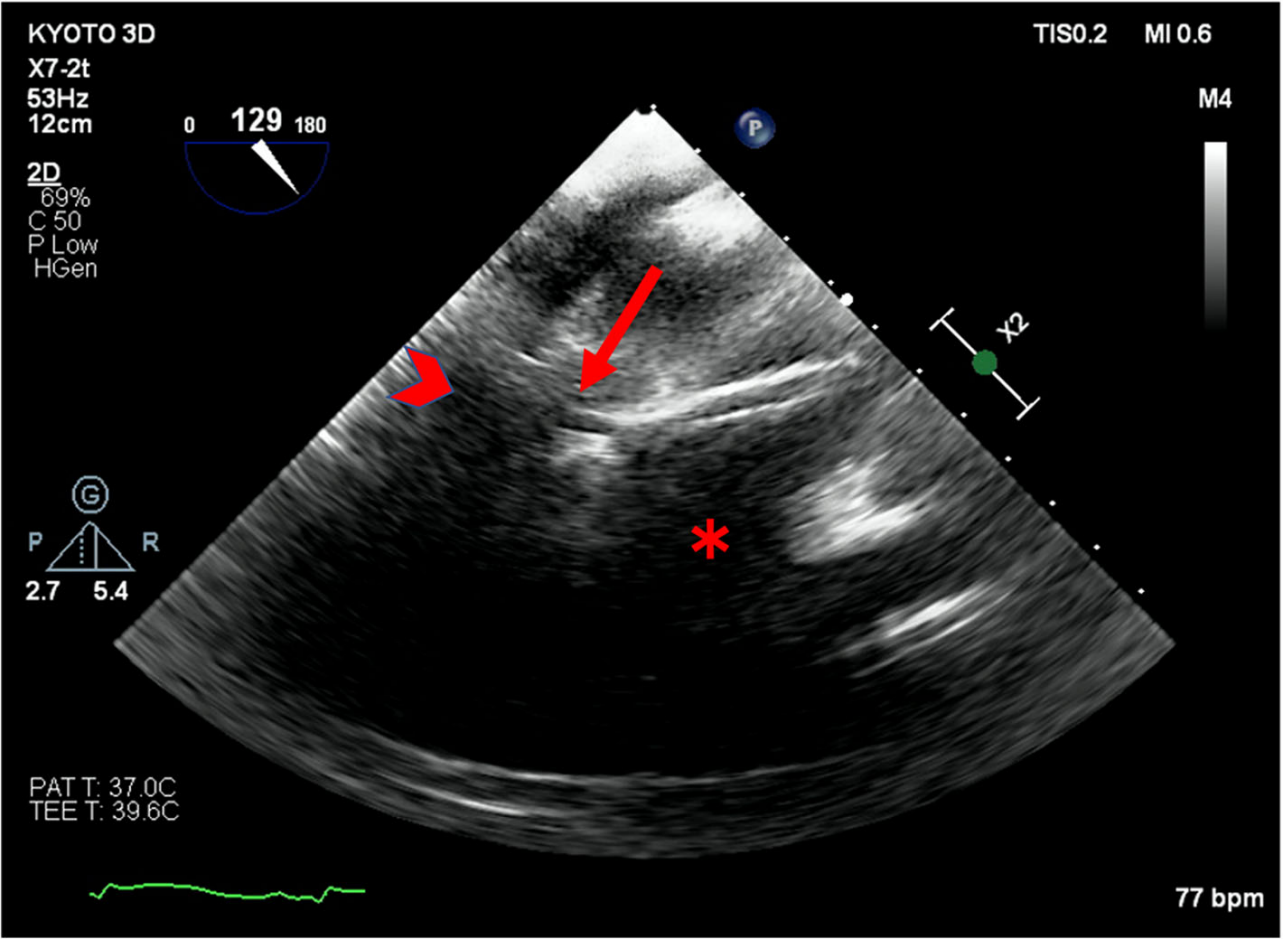
A straying tip of the IMPELLA® in the left ventricle apical wall detected by transgastric long-axis view by transesophageal echocardiography. Note the massive pericardial effusion (arrow head) and the two highly echogenic parallel lines indicating the tip of the IMPELLA® and the thinning left ventricular apical wall (small arrow). Left ventricle anteroseptal wall was not depicted probably because of acoustic shadow of the IMPELLA® (asterisk)

The TTE performed after PCI in the angiography room showed increased pericardial effusion and the abnormal flow on apex of the left ventricle (Fig. 1). Although the tip of the IMPELLA® was not clearly depicted by TTE, the insertion of the IMPELLA® might have caused an iatrogenic left ventricular free wall rupture. The patient was transferred to the operating room under PCPS and IMPELLA® support to release the cardiac tamponade and repair the left ventricular wall. After introduction of anesthesia, TEE revealed a large amount of pericardial effusion, and the tip of the IMPELLA® straying into the thinning left ventricle apical wall (Figs. 2 and 3). When the pericardial sac was incised, blood erupted and a large amount of clot was observed on the left ventricular surface. Cardiopulmonary bypass was established and the PCPS and IMPELLA® were stopped. When the clot was removed, hemorrhage was seen from a thinning apical free wall approximately 2.5 cm long on the side of the left ventricular anterior descending branch, and the tip of the IMPELLA® was palpable.

The IMPELLA® was carefully removed and the lesion was sutured for left ventricular wall repair. While PCPS was not functioning well due to poor blood removal, cardiac function improved with the release of cardiac tamponade. Therefore, the PCPS was terminated and the patient was weaned from cardiopulmonary bypass only with a reinserted IABP. The IABP was weaned on postoperative day (POD) 9. Tracheostomy was performed on POD 15, but the patient went into septic shock on POD 34 and died on POD 35.

## Discussion and conclusions

Cardiogenic shock occurs in 5 to 8% of patients hospitalized with ST-elevation myocardial infarction [1]. Despite aggressive treatment modalities such as PCI and use of intra-aortic balloon support, mortality of cardiogenic shock remains at 50–70 % [5]. Recovery of myocardial performance following successful revascularization of the infarct-related artery may require several days [5]. During this period, many patients develop low cardiac output [5], and mechanical circulatory support is used to prevent hemodynamic instability. The IMPELLA® is a minimally invasive assisted circulation device that can be implanted using a catheter technique and has efficacy and safety in patients presenting with cardiogenic shock [6]. The IMPELLA® has been approved by the US Food and Drug Administration for use in patients with cardiogenic shock for up to 6 days and for high-risk coronary interventions for up to 6 h [7, 8].

Guidelines for optimal IMPELLA® placement stipulate that the inlet of the IMPELLA® should be 35 mm below the aortic valve and the outlet should be above the aortic valve [7, 8]. The IMPELLA® is equipped with a position-sensing aperture on top of the discharge, and the position waveform is displayed on a monitor. However, no position waveform abnormality was detected in this case, and Kaki et al. emphasized the importance of using imaging modalities such as TTE and TEE to check for abnormalities in IMPELLA® position [6]. Frequent complications with IMPELLA® utilization include acute limb ischemia, insertion site bleeding, hemolysis, and vascular complications [4]. Left ventricular perforation by an IMPELLA® has occurred in 25 of 407 patients (6.1%) based on the MAUDE database [9]. However, only three cases have been reported worldwide [2–4], and only one of them used TEE. In this case, TEE showed that IMPELLA® inlet was not visible and the entire IMPELLA® was implanted 4–4.5 cm deeper than the aortic valve level [2], which played an important role in detecting the abnormal position of IMPELLA®.

In the setting of transmural infarct, there is a particular need for caution regarding complications following IMPELLA® placement. The histopathologic sequence after ST-elevation myocardial infarction is well characterized and any ventricular instrumentation poses a higher risk than in a normal setting [4]. The possibility of IMPELLA®-induced left ventricular perforation should be considered if new pericardial effusion is detected, the positional waveform of the IMPELLA® monitor changes, or heart failure progresses rapidly [2]. In the present case, TTE showed increased pericardial fluid and findings suggestive of free wall rupture. It was difficult to identify the precise perforation site on TTE, but TEE showed that the tip of the IMPELLA® had strayed into the apical wall of the left ventricle. TEE was also useful in identifying the perforation site.

The IMPELLA® tip has an inlet slightly in front of the tip and a pigtail catheter protruding from the tip [10]. In the present case, an IMPELLA CP® was used, with a pigtail catheter of 6 Fr and a cannula of up to 14 Fr at maximum. Two highly echogenic parallel lines that appeared to be the IMPELLA® tip strayed into the apical wall of the left ventricle based on TEE findings. On TEE, it was not possible to distinguish the difference between the pigtail catheter and the cannula. The tip was not visible from the surface of the heart and was only palpable at a point on the free wall where bleeding and thinning were observed. This suggests that only the tip of the pigtail catheter may have perforated the left ventricular free wall.

The perforation site coincided with the dominant region of the left anterior descending branch #7, which was completely occluded, suggesting that left ventricular perforation was caused by weakening of the myocardium. If left ventricular perforation by an IMPELLA® is suspected, the IMPELLA® should not be repositioned or removed immediately to avoid catastrophic bleeding [4]. Under cardiopulmonary bypass, the site of perforation should be confirmed and the IMPELLA® should then be carefully removed. It is not clear when left ventricular perforation occurred in the present case. In a past report, the IMPELLA® migrated deep into the left ventricle and subsequently eroded through the wall during transportation of the patient [2]. However, the report did not include any information on the implantation procedure or postprocedural monitoring at the referring center. Ventricular perforation by a 6-Fr end-hole catheter, such as the multipurpose catheter used for indwelling, is common [11].

In our case, PCI under PCPS and IABP support was performed for cardiogenic shock, but due to prolonged hypotension, the IABP was removed and an IMPELLA® was placed instead. TEE showed that the tip of the IMPELLA® strayed into the apical wall of the left ventricle, and TEE was useful for identifying the site of perforation. The tip of the IMPELLA® must be implanted in the left ventricle, but the fragile left ventricular wall due to infarction can be easily perforated. It is extremely important for us anesthesiologists to keep track of the proper placement of the IMPELLA® at all times via various monitors, including TEE, in anticipation of situations such as this case.

## Data Availability

Data for this case report are unavailable for public access because of patient privacy concerns.

## Abbreviations

TEE: Transesophageal echocardiography
TTE: Transthoracic echocardiography
IABP: Intra-aortic balloon pump
PCPS: Percutaneous cardiopulmonary support
PCI: Percutaneous coronary intervention
POD: Postoperative day
